# Trust and Privacy Solutions Based on Holistic Service Requirements

**DOI:** 10.3390/s16010016

**Published:** 2015-12-24

**Authors:** José Antonio Sánchez Alcón, Lourdes López, José-Fernán Martínez, Gregorio Rubio Cifuentes

**Affiliations:** Centro de Investigación en Tecnologías Software y Sistemas Multimedia para la Sostenibilidad (CITSEM), Campus Sur Universidad Politécnica de Madrid (UPM), Ctra. de Valencia, km. 7. 28031 Madrid, Spain; lourdes.lopez@upm.es (L.L.); jf.martinez@upm.es (J.-F.M.); gregorio.rubio@upm.es (G.R.C.)

**Keywords:** Smart Cities, Smart Grid, Internet of Things, Wireless Sensor Network, security services, privacy, personal data protection, Utility Matrix

## Abstract

The products and services designed for Smart Cities provide the necessary tools to improve the management of modern cities in a more efficient way. These tools need to gather citizens’ information about their activity, preferences, habits, *etc.* opening up the possibility of tracking them. Thus, privacy and security policies must be developed in order to satisfy and manage the legislative heterogeneity surrounding the services provided and comply with the laws of the country where they are provided. This paper presents one of the possible solutions to manage this heterogeneity, bearing in mind these types of networks, such as Wireless Sensor Networks, have important resource limitations. A knowledge and ontology management system is proposed to facilitate the collaboration between the business, legal and technological areas. This will ease the implementation of adequate specific security and privacy policies for a given service. All these security and privacy policies are based on the information provided by the deployed platforms and by expert system processing.

## 1. Introduction

A smart city represents a leap forward in increasing a city’s sustainable growth and strengthening city functions to provide a greater quality of life for citizens than a traditional city. It is predicted that there will be a great quantity of “*objects*” interacting continuously with citizens and which can be both collectors and distributors of information regarding their mobility, energy consumption, *etc.* As a result, cyber and real worlds are strongly linked in a smart city. Thus those “*objects*” can act as sensors and actuators to interact with the smart city [1,2]. New services based on information gathered and recorded from multiple sources can be deployed when needed. The loss of trust and privacy of citizens could be an obstacle in the interaction between smart city and citizens. Citizens with their mobile phones and other smart devices, such as wearable devices, can also act as sensors, and they can give information about their movements, habits, preferences, *etc.* One of the most significant perceived risks for citizens is the tracking of their movements and their activities through the information gathered by the objects. They also fear being included in a list of personal profiles. Analyzing these data in order to identify behaviors and habits of people, yields information that could be used in many areas, mainly in marketing.

The set of services in a smart city can be viewed as a holistic compound service comprising all single services such as urban mobility, energy consumption, critical infrastructures, public safety, health *etc.* As a result, using the appropriate Smart City technologies, sustainable management of the whole is made possible. There are many stakeholders in a Smart City, each one having their own interests. Among the major stakeholders are sponsors, services operators, and the monitored entities (some of which may be citizens). Stakeholders’ interests may conflict with each other. The solution of these situations represents a challenge for legislative and regulatory entities. Therefore it is necessary to implement coherent trust and privacy policies based on legislation, since they are able to reconcile the rights and interests of all stakeholders and protect the citizens from infringements of their rights and invasions of their privacy. Nevertheless, this new range of services also requires the development of new communication architectures to minimize their vulnerability and ensure the maximum protection to citizens. Therefore it is necessary to develop and research about new mechanisms to provide safe and reliable environments. Concepts such as “*Privacy by Design (PBD)*” [3] and the mechanisms to facilitate positive or negative consent are being researched in order to build confidence and allow the users to choose. This idea also requires the participation of actors and stakeholders to protect against the possible chaos if a mass deployment of these technologies were to occur. Thus, for the *PBD* seven principles are defined [3], these deal with proactivity; prevention; privacy settings configured by default and integrated into the design, *etc.* They also deal with the visibility, transparency and designs needed to focus on the user and respect people’s privacy.

This paper proposes two goals: (1) A platform to integrate the functionalities and control of the services to acquire enough capacity to generate new applications; (2) An expert system to solve the diverse legislation issues and provide options to generate a policy of trust and privacy mechanisms to apply.

Currently, several *Interconnection and Cooperation Platforms* (*ICP*) are being developed. These platforms also have to allow management of trust and privacy policies. One of them is the “*ACCUS Project* [4]”. The Adaptive Cooperative Control in Urban (sub)Systems (ACCUS) platform aims to implement three innovations: (1) integration and coordination platform for urban systems; (2) new control architecture for urban subsystems and (3) general methodologies and tools for creating applications.

This paper is organized in the following manner: Section 2 shows existing related work in this research field, Section 3 shows an brief overview on ACCUS platform in the smart city, Section 4 discusses the major challenges to privacy and trust in that environment. Section 5 shows the needed elements for privacy and trust policy implementation, and in Section 6 these are applied to an example of a Smart Service in a smart city. Section 7 concludes with a summary of the major contributions of this paper and future work.

## 2. Related Work

The 2012 during our investigation activities about security and privacy in the “Internet of Things” field we were able to verify the huge interdependence between the selection of the mechanisms and the security and privacy countermeasures, the right legislation that must be applied to a determined IoT service and the commercial need for the cost to be as low as reasonably possible.

After a study on the state of the art about this topic reported in previous publications [5,6], three different kinds of contributions were found: (1) state of the art regarding commercial products and businesses focused on the creation of new ideas and services to be commercialized; (2) technology-based state of the art, which provides better and more efficient solutions by its natural progress; (3) legislative state of the art, which is not always homogeneous for the different markets where it is expected to be used, and with a very significant impact on the companies related to the sectors where they perform their activities. The timing that is required for each of the groups is very different and they do not always move at the same speed, thus resulting in potential risks for people, critical infrastructures, *etc.*

The study that was carried out started by analysing the selection process of the security and privacy mechanisms that were made during the specification and design process of several products and services made by two relevant companies of the sector. These companies, although unwilling to be identified in this manuscript, nevertheless provided us their support. Mechanisms were selected according to the technological solution suitable to the legal requirements that were obtained by means of the counsel requested to consultancy companies regarded as leaders of legislative cases involving Internet usage. In this process we have found a significant amount of issues. Several of them have been enumerated as follows:

1. Consultancy costs are high, both in economic and time-to-market terms.
2. In some cases, costs associated to security made the product or the service unviable, resulting in the cancellation of the service after significant resource expenses, or the redesign of the service, thus increasing the related costs.
3. Concern was manifested by the companies consulted with regards to the associated cost of claims, complaints, sanctions and corporative image deterioration that suppose the impacts tied to security and privacy flaws.
4. Both companies pointed out the convenience of having a simulator to test new ideas for products and services that could provide them with a forecast of the requirements for security and data protection in order to perform a cost evaluation prior to the start of the development phase.
5. Heterogeneity of legislative frameworks in the different countries those companies operate in is a major issue for them.
6. This heterogeneous legislative framework also affects the same countries or even the same service, depending on how it is used [5,6]. This also happens when one product is designed as a combination of some others, when the same terminal is used to offer several services, *etc.*
7. Actions to be taken in a context of likely legislative changes, or when dealing with emergency level changes that may imply different features related to security and privacy.
8. Previous knowledge of the impact that a specific legislative change will have (in terms of security and data protection on the IoT-based products and services already deployed) is prone to be helpful for the agents involved in legislation.
9. The user tends to recklessly offer his/her trust less often. What he/she really requests is be guaranteed that their information, intimacy, security and safety will not be jeopardized by the mere fact of voluntarily using (or refusing to use) these new products and services.

After several months of study and consultations to the members of the “Internet Society” related to the legal area of knowledge, political parties, trade unions, *etc.*, the idea of having these three areas of knowledge cooperating (business, law and technology) with each other started to build up. This concept of channelling the requirements of security and privacy through a collaborative system among the areas of business, law and technology is an original contribution the authors of this manuscript (this idea has been contrasted and validated by the interlocutors previously mentioned).

This collaboration materializes itself in the collection of the entrepreneurial, legislative and technological knowledge that can be used by an expert system to provide an answer for the already mentioned main challenges.

We couldn´t find any system or integrated packet that would adapt to the automation that we were looking for. The closest were the “Legal Expert Systems”, even though analysis and legislative conflicts were their focus. The expert system proposed in this paper was inspired basically on Cuadrado Gamarra´s book about expert systems in the legal field [7], and the articles of Stevens [8] and Venkateswarlu [9] as well as related references in these about “Legal Expert Systems”. Within our paper the vision is a bit different; we do not try to solve legislative conflicts as is the case of the previously mentioned works [7,8,9], but rather what we want is to obtain the key legislative knowledge needed in a matter of security and privacy to be able to apply the needed legal imperatives, about a concrete IoT service.

On the other hand and due to the large researcher activity about it, a lot of investigation studies are available (among them the major part of the bibliography in the previous publications [5,6] mentioned before in this paper) providing mechanisms and technological countermeasures to act against the threats and attacks to the security and privacy being able to provide solutions.

Therefore, knowing the details of the IoT service that is to be developed, the legal imperatives that must be applied and a group of available technological solutions, it should be possible to manage a solution tailored for each situation. This is the purpose of the proposed expert system.

The structure of the system relies on not hindering the independent evolution of each of the spheres, each of them with its own budget capabilities, Information Technologies systems and their own route map for their own progress. The only adaptations that must be done are involving data communication, transfer and results storage; all the other actions can be performed in each of the spheres with their regular means of work.

This paper proposes an expert system to generate security and privacy policies for services in the smart city. This policy is communicated to the ACCUS platform, which is able to deploy it to the network and devices. The expert system proposed in this research has gone though various major versions since its first design. The first version was presented at the “Third Intech Conference in London, 2013” [5]. The first version was designed to decide the security level that is needed for a certain use case for a specific service. It managed the behavior for different use cases using the same WSN dedicated to health monitoring, but subjected to different legal frameworks. In that case the expert system provided its security and privacy policies to a service platform called AWARE which was then able to configure the WSN remotely.

The second version was designed in 2014, when its functionalities were expanded and the model was modified to be able to work with more than one WSN in different technologies or IoT services [6]. Thus, the expert system was improved to be able to manage the requirements of various services, taking into account the possibilities of different technologies. At the same time, the platform mentioned before was boosted to be able to communicate and configure the security mechanisms for various WSN technologies.

Lastly, this paper wishes to further expand the model of the expert system to be capable of selecting the different security and privacy levels for each one of the services in a smart city. Each newly generated services and created by combination of the existing ones must have a security level adequate, maybe can be different than the ones being used. It also offers the possibility of changing the security level in a city, depending on the possible states of alarm or emergency. In this environment, the mediating platform for the city’s services is ACCUS [4]. This research paper provides a way to tackle the issues and challenges with regards to security and privacy in the Internet of Things within the framework of a smart city. These challenges have a major impact in the entrepreneurial, legislative and technological environments, and while each of them offers only one part of the solution, the final solution must come from the collaboration among all three areas. Another important issue is that flaws in security and privacy may affect people´s rights.

This paper does not propose any legislation framework and no security mechanism, but rather it describes a method to choose and apply the security services based on the collaborative environment among the business, legal and technological areas.

## 3. Smart Cities Applications and Urban Systems Management Using ICP

### 3.1. Smart City Applications

Smart city applications are grouped into several areas. One classification is proposed in [10], based on the presence of six characteristics shown in Table 1. All these areas raise new challenges in security and privacy such as transnational authentication systems for citizens and businesses, agreed frameworks for data privacy, and the sharing and collection of individual and business data, in order to make a more livable city for citizens, the performance of integrated services and urban systems (such as manager of the traffic, energy, lighting, emergency systems, or information systems) must be taken into account. This enables integrated management strengthened through mutual aid in situations that require it.

**Table 1:** Smart City applications.

Applications	Target
Smart Economy	Innovative spirit; Entrepreneurship; Economic image/trademarks; Productivity; Flexibility of labor market; International embeddedness.
Smart People	Level of qualification; Affinity to lifelong learning; Social and ethnic plurality; Flexibility; Creativity; Cosmopolitanism/Open-mindedness; Participation in public life.
Smart Governance	Participation in decision-making; Public and social services; Transparent governance; Political strategies/perspectives
Smart Mobility	Local accessibility; Accessibility; Availability of ICT-infrastructure; Sustainable, innovative and safe transport systems
Smart Environment	Natural conditions; Pollution; Environmental protection; Sustainable resource management
Smart Living	Cultural facilities; Health conditions; Individual safety; Housing quality; Education facilities; Social cohesion

For example, correct traffic management in emergency situations can contribute to emergency services arriving in the shortest possible time wherever they are required, and could also reinforce or restrict other resources in the same area, *etc.* To obtain this range of new applications, it is necessary to integrate both the performance and control of these autonomous systems. It should be noted that the systems providing specific services must continue to evolve independently and their integration with other systems must not impede their natural path of evolution, but must find a way that does not affect their integration with others, in a scenario of an integration of “*systems of systems*”. Each system has its own internal evolution, which must not be affected by the integration process, so an integration platform that enables the possibility for each platform to maintain its functionality and control would be necessary, and to obtain with the integration the additional advantage of having enough capacity to enable the generation of new applications.

### 3.2. ACCUS Project

The proposal in this paper has been deployed inside the European ACCUS project, but the proposed expert system is in fact adaptable to any other platform that can control, send and receive commands and responses to/from network elements and perform the needed remote configurations.

As indicated in [4] the ACCUS project focuses on four innovations that are listed below:

- Provide an integration and coordination platform for urban systems to build new applications across urban systems.
- Provide adaptive and cooperative control architectures and the corresponding algorithms for urban subsystems in order to optimize their combined performance.
- Provide general methodologies and tools for creating real-time collaborative applications for “systems of systems”.
- Seamless connectivity and semantic interoperability among all services and subsystems connected. ACCUS ICP must provide the necessary mechanisms and facilities so that present and future applications and services connected within the smart city can consult which other subsystems and services exist and what is their functionality.

**Figure 1:**
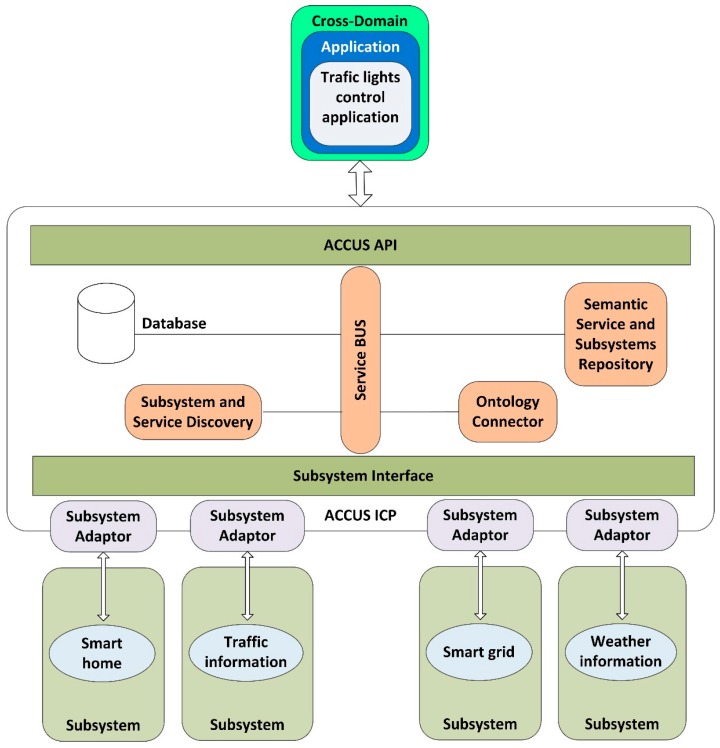
Registration and discovery of subsystems and services in ACCUS.

Currently, the platform has two types of components: (1) core components: the components which allow the platform to provide its basic functionality such as registration, discovery, control elements, security, *etc.* and (2) city customization components: in order to enable the adaptation of the ICP platform to any city, it must have some plugins that allow this customization, e.g., event detection, location detection, data analytics, situation awareness… A main functionality of the ICP platform is to provide the registration and discovery of the provided services by the subsystems. Figure 1 shows the basic outline of registration and discovery of the subsystems and services. In each record, the subsystems or services must be recorded semantically according to the platform semantics. In case the semantics of the service does not match, the adapter subsystem must perform the semantic conversions needed.

The components are connected to a service bus to exchange the messages that allow them to interoperate. The functionality of each component of ICP is:

- Service bus: provides the interconnection and cooperation of the component based on a paradigm of message exchange. It could be implemented using already existing products such as *JBOSS* [11] or *WSO2* [12].
- Subsystem and service discovery: discovers all subsystems connected to the ICP and the services provided by each subsystem. This component works in real time. It is responsible for registering subsystems in the semantic repository.
- Ontology connector: handles the translations of the data which must be added to the Semantic Repository, when these data are in a known XML format [13,14]. These translations from XMLs to RDFs are done using a previously generated mapping file, which describes, what elements/data from the source XML, must be stored as instances of classes of the ACCUS Ontology. A mapping file must be defined per each type of XML
- Semantic Service and Subsystem Repository: stores the semantic description, in a way that complies with the proper ontology of services and subsystems registered in the ICP. When a new subsystem or service is discovered their semantic description must be stored in this component.

**Figure 2:**
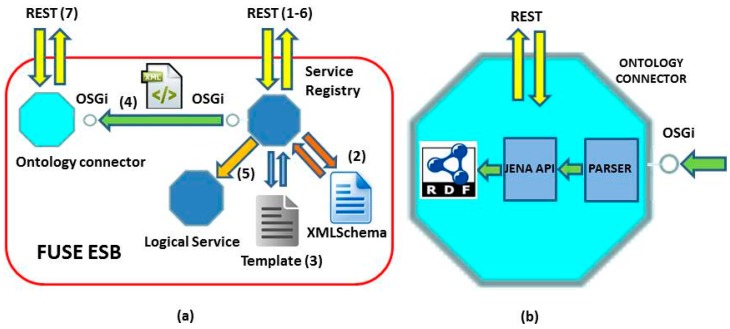
(**a**) Overall layout; (**b**) Ontology connector.

Currently, ACCUS’s architecture is in the development and demonstration phase. However, it has been evolved from an architecture developed and tested in the e-Gotham project [15] as it is shown in [16]. Thus, the architecture presented here has been partially developed and tested. A new service is registered via the following procedure as shown in Figure 2a:

(1) A request is sent to register a new service. The service previously has connected to ESB. It could be REST service, Web service or any of supported by the ESB.
(2) The request is validated against an XMLSchema so as to check whether there is any issue with the request. If the request in sot valid, will be rejected.
(3) A template can be filled with the mandatory information in XML format (semantic or non-semantic format). Of a set of templates, the most appropriate will be chosen.
(4) An XML file is sent to the Ontology Connector (Figure 2b) via OSGi interfaces.
(5) A Logical Service is created in real-time based on an Archetype. This is a key functionality since it allow to have a registrable version of the physical service.
(6) Logical service registry is acknowledged.
(7) The status of the registry can be requested via the ontology connector (Figure 2b); semantic, rdf-based responses will be obtained. To do this we use Jena API in order to build a java version of the ontology, and execute the set of parser on the ontology, let, in this way, ho manage a semantic repository in RDF.

**Figure 3:**
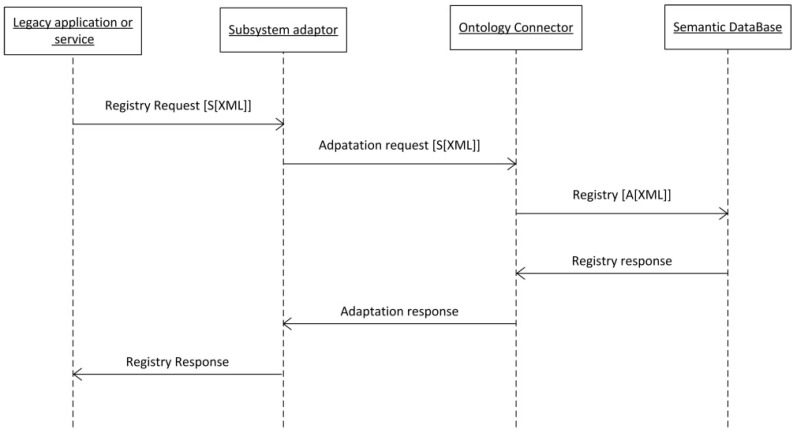
Registration of a service in ACCUS.

In Figure 3 the sequence diagram that specifies the registration process of a service is shown. When applications need to know the services to be used or included, a query must be sent to the Semantic Service and Subsystem Repository. The response to this query will be a set of XML files with the profile of the services available at a given moment.

Since the goal of the platform is to generate semantic interoperability for seamless connectivity, all the agents listed must be semantically annotated in the same way, so that a service or application can request a specific query to the Semantic Service and Subsystem Repository, and then get a reply that complies with known semantics, in this case, the ACCUS ontology. This ontology integrates the meaning of all the components of the ACCUS platform (core and city customization), the sensors and actuators, (e.g., SSN) [17], people (e.g., FOAF) [18], city model (e.g., cityGML) [19], services and subsystems in the city (for the smart grid subsystem, an *ad-hoc* ontology was developed in the e-Gotham project [16]).

The subsystems and services may be semantically annotated according to the ACCUS ontology, their own, or none whatsoever, but the ACCUS ICP must provide, as a response to the queries received, results in accordance with the ACCUS ontology. To do so, the Ontology Connector component performs the necessary transformations so that the registry in the Semantic Service and Subsystem Repository complies with the ACCUS ontology, as seen in Figure 3.

The legacy application or service does not have to know the ACCUS ontology, it will send the query with its own annotation format [S[XML]] (previously known by Ontology Connector) and with its own protocol. If necessary, a transformation protocol will be done in the Subsystem Adaptor in order to provide to the Ontology Connector the request of the legacy application or service in the field set in the communication protocol between Subsystem Adaptor and Ontology Connector. Once received the request in the original formal, it will execute the suitable transformation to ACCUS ontology syntax [A[XML]]. In this way, registration will occur in the Semantic Database. The response it will be sent to the Ontology Connector, which if necessary will transform it from ACCUS ontology syntax to Legacy Application or service syntax and Subsystem Adaptor will do the appropriate protocol transformation changes and it will be sent to the originator of the request. An example of a registration for a smart home, consisting of enhanced tele-assistance at home, can be depicted as follows:

<?xml version="1.0" encoding="UTF-8"?>
<service>
    <profile>
              <serviceIdentification>
                       EN_TEL_ASSIST_1
              </serviceIdentification>
              <functionality>
                       <preconditionDescription>
                                service on
                       </preconditionDescription>
                       <outputDescription>
                                Celsius degrees float
                       </outputDescription>
                       <outputDescription>
                                CO2 level integer
                       </outputDescription>
                       <outputDescription>
                                Smoke presence binary
                       </outputDescription>
              </functionality>
              <security>
                       <policy>basic security policy</policy>
                       <dataProtection>integrity</dataProtection>
                       <dataProtection>autehentication</dataProtection>
					   
              </security>
              <grounding>
                       <inputMessage>
                                start
                       </inputMessage>
                       <outputMessage>
                                sensorID-lenghtMessage-PreviousValue-CurrentValue
                       </outputMessage>
                       <endPoint>
                                /icp/assist/home1
                       </endPoint>
              </grounding>
    </profile>
    <process>
              <processID> </processID>
              <typeOfProcess>
                       <atomicProcess/>
              </typeOfProcess>
              <operations>
                       <operation id="read">
                                <preconditions>
                                          device on service on
                                </preconditions>
                                <insANDouts>
                                          <output>float</output>
                                          <output>integer</output>
                                          <output>binary</output>
										  
                                </insANDouts>
                       </operation>
              </operations>
    </process>
    <context>
              <serviceType>
                       <loction> indoor</loction>
                       <motion>static</motion>
              </serviceType>
              <geoCoordinates>
                       <longitude> 40.33889</longitude>
                       <latitude>3.628611</latitude>
              </geoCoordinates>
              <smartSpace> smart Home 1</smartSpace>
    </context>
</service>


**Figure 4:**
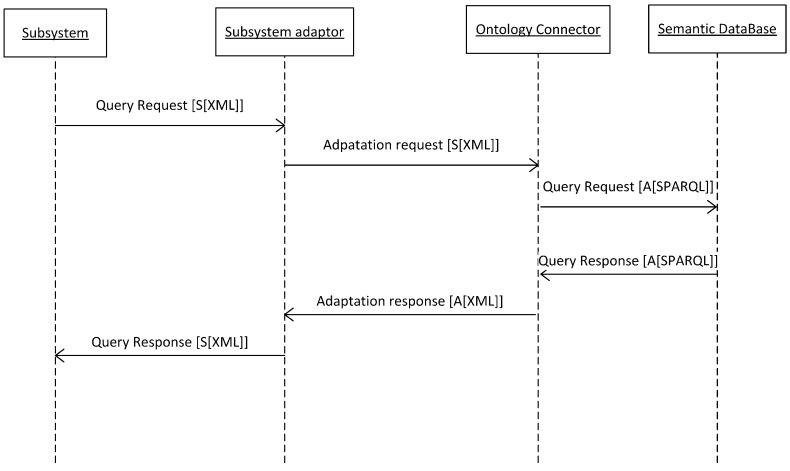
Discovery process in ACCUS.

But, what is the real utility of ontology in this process? Since the main purpose of ontology is to represent in a standard way the meaning of contents with the goal of inferring new knowledge, semantic interoperability allows access to everything registered within the ACCUS ICP, in accordance with the ACCUS ontology. This approach enables a new service or application to send a query to the Semantic Service and Subsystem Repository in SPARQL [20], and obtain as a result information about other subsystems, services; components or devices are connected within the Smart City, their function, and form of access. Since this is a repository in RDF [21], an appropriate response will be sent back to the agent that performed the query. The discovery process of the registered services is shown in the next sequence diagram (Figure 4).

If, for example, an application is willing to become aware of certain features of the services registered for selecting the more suitable one, it will generate a query that may be SPARQL-formatted or not. If necessary, it will be ported to SPARQL by the Ontology Connector module and executed in the Semantic Registry:

PREFIX ns:<http://www.semanticweb.org/ACCUS/1.1#>
SELECT ?ServiceIdentification ?inputDescription ?outputDescription ?policy ?dataProtection ?endPoint
WHERE {
    ?service ns:hasProfile ?profile.
    ?profile ns:hasServiceIdentification ?serviceIdentification.
    ?profile ns:hasFunctionality ?functionality.
    ?profile ns:hasSecurity ?security.
    ?profile ns:hasGrounding ?grounding.
    ?functionality ns:hasInputDescription ?inputDescription.
    ?functionality ns:hasOutputDescription ?outputDescription.    
    ?security ns:hasPolicy ?ns:policy.
    ?security ns:hasDataProtection ?dataProtection.
    ?grounding ns:hasEndPoinf ?endPoint.
}


Response will be in an XML-formatted message according to the ontology:

<?xml version="1.0" encoding="UTF-8"?>
<services>
<service>
    <profile>
              <serviceIdentification>
                       EN_TEL_ASSIST_1
              </serviceIdentification>
              <functionality>
                       <preconditionDescription>
                                service on
                       </preconditionDescription>
                       <outputDescription>
                                Celsius degrees float
                       </outputDescription>
                       <outputDescription>
                                CO2 level integer
                       </outputDescription>
                       <outputDescription>
                                Smoke Presence binary
                       </outputDescription>
              </functionality>
              <security>
                       <policy>basic security policy</policy>
                       <dataProtection>integrity</dataProtection>
              </security>
              <grounding>
                       <endPoint>
                                /icp/assist/home1
                       </endPoint>
              </grounding>
    </profile>
</service>
<service>.....</service>
<service>.....</service>
</services>


With the information therein it will be capable of inferring new knowledge that, in the context of a smart city, consists of generating new cross-domain applications and services for the city and citizens, which then must also be registered in the ACCUS ICP, producing constant feedback. Furthermore, these new applications will already use the ACCUS ontology.

### 3.3. Security and Privacy in a Smart City

Studies performed some years ago such as [22] recognized the importance of data privacy and personal identity among the aspects to be dealt with, not only on technical grounds, but also concerning legal frameworks:

E-Government: There are a number of technologies that will be required for the underlying infrastructure that is needed to help support this process. Fundamental technologies are key to the development of the Digital Single Market (such as authentication and privacy), and to the development of e-government in smart cities. The development of transnational authentication systems for citizens and businesses, the development of agreed frameworks for data privacy, and the sharing and collection of individual and business data, must be considered.

Health, Inclusion and Assisted Living: The key technical requirements to be addressed in this domain are: security (encryption, authentication and authorization), service discovery, scalability and survivability, persistence, interworking, community-to-community application messaging propagation, auditing and logging, location information sharing, and application service migration.

The challenge related to ICT security aspects has to be ensured by a manageable access control management system, to ensure that only authorized persons are allowed to access the data, and ensures that the data is protected to achieve confidentiality. Users should manage authorization. Dedicated authentication and logging mechanisms have to support the enforcement of access control. The challenge in this approach is that access control architecture has to enable both the decentralized storage of data, and the comprehensive access control mechanisms and enforcement that concern all parties that could have access to that data.

Intelligent Transportation Systems: the provisioning of flexible, scalable and self-optimized networks, dealing with heterogeneity, effectively exploiting location information, guaranteeing real-time exchange of data where needed, and providing security, privacy and authentication mechanisms.

Smart Grids, Energy Efficiency, and the Environment: Other challenges include: new communication and networking ICT technologies, new affordable devices that gather environment data, new intelligent algorithms for smart ubiquitous environments, new light sources, new and fair regulations that enables the mass implementation of the Intelligent Street Lighting System idea provided by different vendors; new products for global markets that enable steady economic growth; and advanced products and services based on IP to foster innovations, and economic growth based on an open innovation scheme. Recently, Sicari *et al.* presented in [23] a vision of the near future in security, privacy and trust in IoT. Finally, Weber *et al.* presented in [24] the forthcoming issues in privacy applied to IoT.

## 4. Challenges on Privacy and Trust

It is important to know how to classify data sources in a smart city and their relation to personal identity. These sources are the following:

- Non personal sources: data, unrelated to specific people, gathered from devices (temperature or humidity sensors, *etc.*)
- Personal sources: data, related to specific people, gathered from devices, unambiguously using user identity (social networks, *etc.*)
- Anonymous sources: data related to specific people gathered from devices, but which have been pre-processed to mask their personal identity (covering faces in video camera images, *etc.*).

It may be possible to discover user information by processing data from several sources. These situations must be considered. From a technological viewpoint the security and privacy problems can be grouped as follows: (1) Problems related to computer security and communication systems; (2) Problems related to database security, user identities and communications; (3) Problems occurring when new subsystems are added to the smart city (increasing its complexity and vulnerabilities).

### 4.1. Problems Related to Computer Security and Communication Systems

These are the problems such as malware (viruses, trojans, worms, backdoors, spyware, *etc.*), or bots, loggers, rootkits, DDoS attacks, lack of updates [25,26], *etc.* They are prevented by installing suitable antivirus, firewalls, honeypots, intrusion detection systems (IDS), security policies, updating and implementing system authentication measures. When the devices are localized all around the city and do not have a common control platform, updating or implementing the new policies of authentication or refusing the authorizations is difficult.

### 4.2. Problems Related to Database Security, User Identities and Communications

- Database security [27] The Statistical Disclosure Control (SDC) techniques consist of inserting noise or aggregations to maintain privacy, while maintaining the significant value of the data. Private Information Retrieval (PIR) techniques are based on queries asking for more than the necessary information in order to hide the specific information demanded by the user.
- To hide user identities accessing location-based services (LBS) techniques are used such as cloaking and using pseudonyms.
- Privacy in communications, advanced cryptography and access control can be used to prevent eavesdropping on the data and prevent unauthorized connection nodes to the networks with distributed devices in access public places [28].

### 4.3. Problems Occurring When New Subsystems Are Added to the Smart City

When new subsystems are added to the smart city the complexity and the number of vulnerabilities grow [28], which can be exploited by malicious people to harm the most vulnerable systems and enter into the other subsystems of the smart city.

- Increased interconnections among services increase the ways through which a virus can propagate. Hackers can move through the interconnections among the systems and take control.
- Dependencies among infrastructures. A failure in one of the nodes in the dependencies network could cause some cascade problems. Planning and management can alleviate the problem [29].
- The connection of the smart city with the other platforms and applications by middleware is a strategic element. Those connections must be secured, implementing confidentiality, integrity and authenticity and they must also be interoperable.
- The fact that having a great quantity of services and data sources facilitates creating new applications and services, but risks the availability of these services if a fault in any of them occurs, which would cause malfunctions an application and even make it unusable.
- In an open data context with a great quantity of information sources (both real-time and historical), publishing new data makes it difficult to ensure that they cannot be used to infer the identity of users (using correlation techniques,...). To minimize the time of intrusions and attacks, solutions that implement active reactions in a crisis scenario to curb the anomaly [30] are used.

If users believe that a system is insecure or threatening to their privacy, it will not be able to establish itself successfully in the market. Thus, in order to achieve user consent, trust in, and acceptance of smart cities, the integration of security and privacy-preserving mechanisms must be a key concern of future research. New challenges arise in the area of security and privacy, and they can be classified as follows:

Interconnecting systems that serve completely different purposes (traffic control and energy management for example), and thereby create a “system of systems”, increase the complexity of such collaborating systems exponentially. As a result, the number of vulnerabilities in a smart city system will be significantly higher than that of each of its sub-systems. Furthermore, the pure interconnection of two systems might open new attack vectors that have not been considered before, when securing either of the individual systems. Therefore, research into ways of handling the increasing complexity of distributed systems from the security perspective is required, which includes: cost-effective and tamper resistant smart systems or device architectures (crypto and key management for platforms with limited memory and computation); evolutionary trust models for scalable and secure inter-system interaction; comprehensive security policy; self-monitoring and self-protecting systems, as well as development of methods for designing security and privacy into complex and interdependent systems.

The number of users, and the volume and quality of collected data, will also increase with the development of smart cities. When personal data is collected by smart meters, smart phones, smart vehicles, and other types of ubiquitous sensors, privacy becomes all the more important. The challenge is, on the one hand, in the area of identity and privacy management, where, for instance, pseudonymisation must be applied throughout the whole system, in order to separate the data collected about a user from the user’s real identity. On the other hand, security technologies such as advanced encryption, access control, and intelligent data aggregation techniques, must be integrated into all systems in order to reduce the amount of personal data as much as possible, without limiting the quality of service. It is necessary to work towards interoperability of different identity management systems, as well as automatic consideration of user’s preferences. It is necessary to develop also privacy mechanisms which allow users to express their preferences on service quality and data minimization.

### 4.4. The Challenges

All services in the smart city give rise to new security and privacy challenges and although it is not the main selling issue, users implicitly expect that the involved systems are secure and the privacy of users are kept. A successful attack will directly impact the life of people. Thus, if the users deem that the system is not secure or that it threatens their privacy or their rights, they will refuse to use IoT services, and the solution will not be able to be successfully placed in the market. From the user´s point of view, the requirement is to guarantee the protection of their privacy rights. In consequence, protecting the services of smart cities is a primary issue. So, in order to achieve user consent, and acceptance of smart cities, integration of security and privacy-preserving mechanisms must be a key concern of future research. The challenges can be several aspects:

- Handling of the increasing complexity of distributed systems from the security perspective such as the identity and privacy management such as pseudonymisation throughout the whole system, in order to separate the data about a user from its real identity.
- Integration of security technologies into systems such as advanced encryption and access control, and intelligent data aggregation techniques, *etc.*
- The context of smart cities relates to open data business models. It is possible because services become pervasive and ubiquitous and the opening of the databases will become more important.

The most important issue has to be transparency, so the end-user must be know how his/her information is being used, with clear options and secured environments, when providing services that use personal data.

## 5. Implementation of the Solution in the ACCUS Project Environment

**Figure 5:**
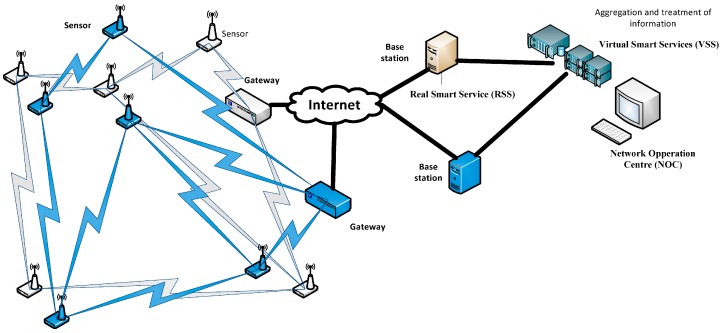
Joint service in the city as the superposition of individual services.

The holistic service in a smart city comprises several components to provide a particular service (smart grid, smart traffic, *etc.*). Each service must have its own security mechanisms to protect itself and the personal data therein. Each particular service has its own security, but the whole service (the joint service) for throughout the city must be considered, since some security holes may exist caused for interactions among those. The whole service in the city from a holistic viewpoint could be handled with some additional security techniques. The joint service in the city as the superposition of individual services is shown in Figure 5. It shows some smart services with a real infrastructure (sensor nodes, communication paths, base station, *etc.*) defined as a *Real Smart Service* (*RSS*), coexisting with other services comprised of combining and processing the information available, defined as *Virtual Smart Services* (*VSS*) [31,32,33]. In this environment the components to protect are sensor nodes, communication paths, base station, and sensible data that flow through them in the *RSS*. Aggregation and information processing must be protected by security mechanism in both *RSS* and *VSS*.

### 5.1. Data Protection Impact Assessment

It is important to note that each particular service is analyzed by a Data Protection Impact Assessment (DPIA) Template [34]. As a result some security holes may appear; therefore making a new DPIA Template for the holistic service could be the solution to fill these holes. A new particular service in a smart city has an effect on itself and on the holistic service, and thus the DPIA is the main tool for continuously reviewing the security mechanisms and countermeasures to satisfy the security and data protection laws. There are some services of IoT such as smart grids for which the legal analysis has already been performed in a DPIA template, but other IoT services have not got them yet. In those cases it must be made by the general method given in [34]. At the end DPIA-T must obtain the Feared events; Threat ID; Related Security & Privacy targets; Affected assets; Impact; Likelihood; Risk Level, that is, the security and privacy imperatives for the entities that must be protected.

**Figure 6:**
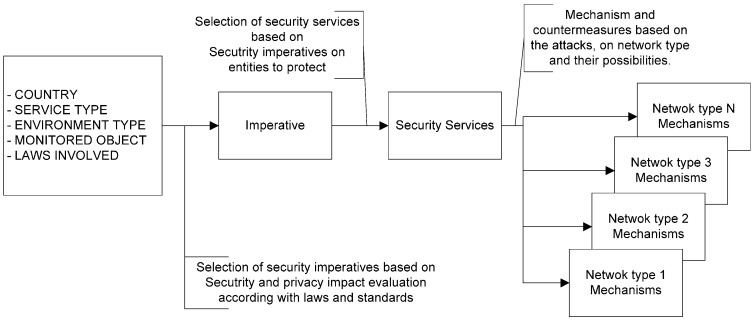
Selection chain of security mechanisms.

### 5.2. Constructing Security and Privacy Policies

As we can see, Figure 6 gives an overview of the selection chain of security mechanisms that constitute a security and privacy policy. To make it, a *DPIA template* is needed, and as a result, the security services are mapped by security imperatives based on the concept. Security services are bound by legal and regulatory frameworks. To comply with them, a *DPIA template* is very useful. Each network type has its own mechanisms and countermeasures, depending on its technology and its limitations (battery, memory, process capacity, *etc.*). A service in the same city made up of different technologies may have different mechanisms to address counterattacks on the same security service (possibly with different results). One *DPIA template* over the holistic service can give supplementary mechanisms sufficient protection. As result of this process some mechanisms and countermeasures could be modified or adjusted.

### 5.3. Automatic Selection System for Making Decisions over Security and Privacy Policies

To advance the state of the art, at first the relevant, available and accessible knowledge in the information resources about security and privacy is analyzed. Useful knowledge flows are singled out, such as researching reports, *etc.*, and what can be done with this knowledge is analyzed. Today there are many studies in highly targeted areas; in fact, in the technical area, there are many studies dedicated to developing new efficient security mechanisms, in order to provide specific solutions to specific cases. In the legal area, the legal implications of this new paradigm are being investigated and some ideas and projects are being developed in this regard. Moreover, companies and suppliers of equipment and networks are also devising services useful to society.

#### 5.3.1. Overview

**Figure 7:**
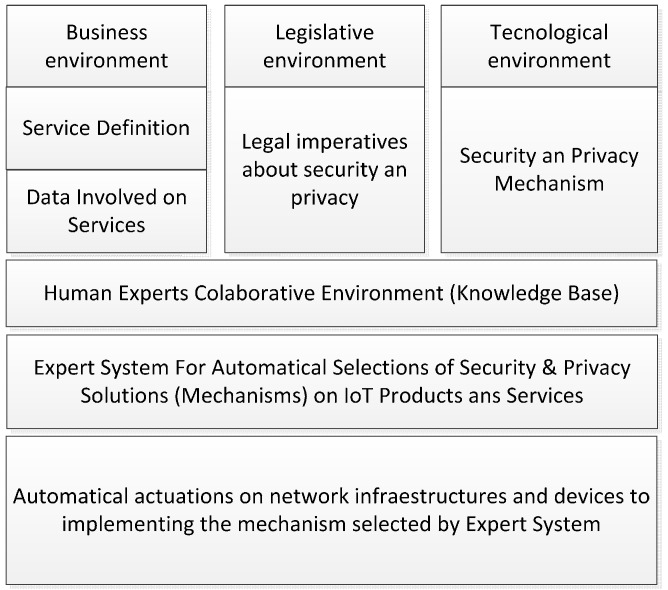
Involved information. Automatic Selection System for making decisions concerning security and privacy policies.

The basic idea of the expert system developed focuses on gathering all this knowledge generated by experts and formalizing it into knowledge bases, making it appropriate for it to be processed to obtain security policies to be applied to products and real services. This idea is developed in [6] and it is represented in Figure 7.

The knowledge generated by the human experts from the areas involved is stored in order to be processed. Data protection measures are selected based on this information network, so the inclusion of these knowledge areas allows for example, to certify to users and corporations that this IoT service is adequately protected.

**Figure 8:**
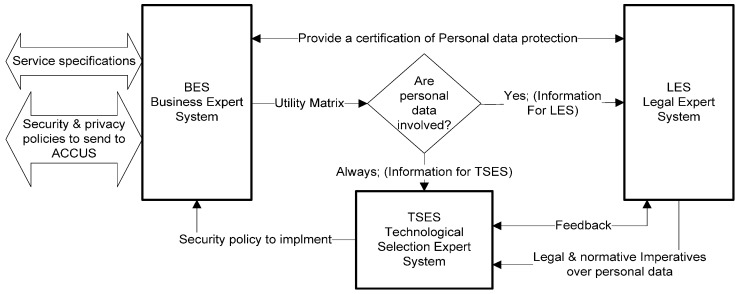
Expert System. Automatic Selection System for making decisions over security and privacy policies.

There are three knowledge bases which are the most important part of the expert system. They contain the results of the collaborative work of the involved areas. These areas are the Business-Business Expert System (BES) about the service definition; Juridical—Legal Expert System (LES) about the Law framework; and the Technological area– Technological Selection Expert System (TSES) about attacks, security services, and mechanisms. The information flow is shown in Figure 8.

In the environment of a smart city it could be very advantageous to concentrate on a *Network Operation Centre (NOC*), the intelligence, maintenance and deployment of actions related to security and privacy policies in the smart city. This way, all this knowledge generated by the different areas can be leveraged, and made available in an information system to act as a support of collaborative work between the areas involved, in order to find the best solutions for the protection of personal data generated in each case.

**Figure 9:**
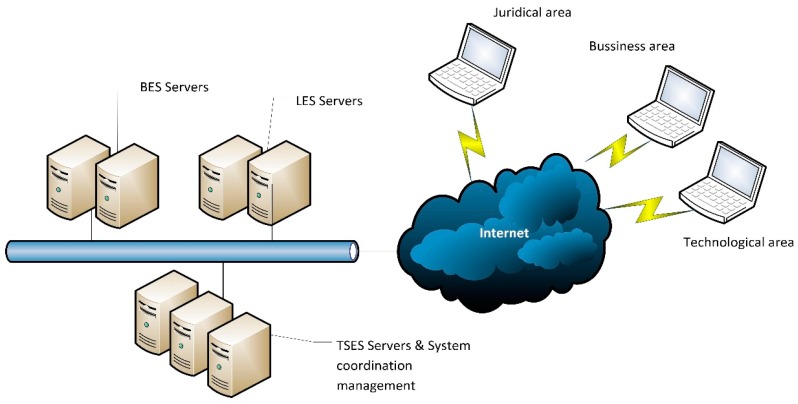
Working network.

The system interacts with the areas that can provide the knowledge and sufficient confidence to provide quality solutions (corporations, legal and technological areas) working network environment. This interaction is represented by Figure 9.

This would allow the issuing of certificates to provide enough confidence for users and businesses. NOC and ICP work together. In an environment like that, companies that want to design new products and services can use the expert system to perform virtual simulations before making decisions over on the actual markets. The legal and political sectors could conduct impact assessments on society and the market about possible changes and new laws on data protection, being able to know how and to what extent existing products and services and future developments would be affected. It would also be useful for the technological sector. It could observe and assess critical aspects that need new research and innovation, or emerging issues that require technological solutions.

The current state of this development performs automatic selection which takes into account the various factors that determine the need for specific services and security mechanisms. These factors include legal and regulatory requirements for personal data protection of the product or service to be provided, its network topology, its physical characteristics, *etc.* All these elements should be considered for a robust implementation of a security system that is able to adapt to each particular case. With these elements, among others mentioned in [35], services and appropriate security mechanisms are chosen to be implemented in the design and construction of the product or service, by a decision based on certain security requirements, which must act over a set of data that must be protected by legal and regulatory requirements.

**Figure 10:**
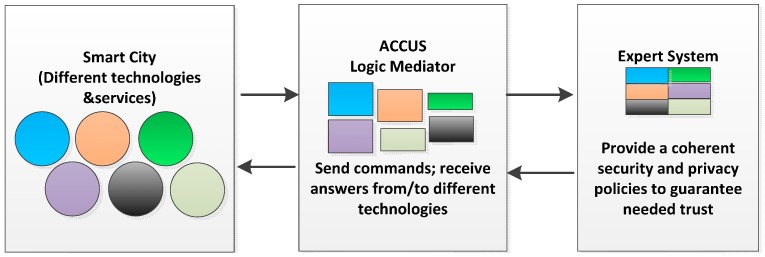
Mediation chain.

When a new service is implemented within the city, or when certain laws have been changed, it might be necessary to implement a new security and privacy policy, or to adapt the exiting one. The expert system described herein decides which policy must be applied in the smart city. Following that, every system and network element involved and distributed within the city must be reconfigured. If these actions are to be performed automatically, ACCUS must act as a mediator, that is, it must be able to translate the new security and privacy policy, received from the expert system, and it needs to generate the necessary commands and actions, adapted to the requirements of each technology of the smart city. This way, suitable mechanisms will be activated according to the security and privacy policy that each service needs to fulfill. This task of configuring and reconfiguring the security and privacy policy of the smart city is accomplished by using the chain of mediation shown in Figure 10.

This figure shows the mediation chain from the expert system to provide coherent policies to the network elements in the smart city with ACCUS as mediator to manage different technologies. The different colors represent different technologies coexisting in the same environment and managed by the ACCUS platform. The expert system also must record the basic security features and privacy of each used technologies.

**Figure 11:**
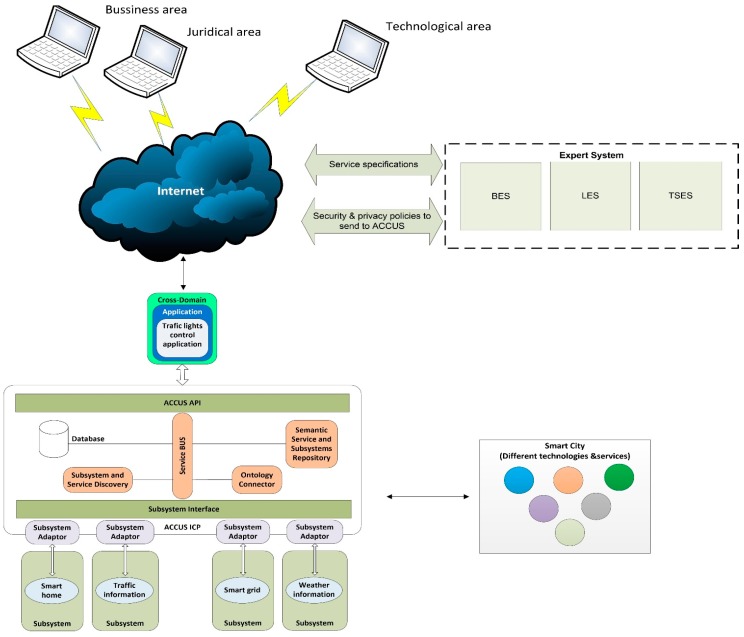
Overview of the system operation.

Figure 11 shows an outline of the interworking between the collaborative environment that generates the required knowledge and interacts with the expert system and the ACCUS platform, which understands and maintains a dialogue with the systems and elements in the city. Each of the areas involved can use the expert system. The expert system can communicate through the areas or communicate the selected policies to ACCUS to generate the necessary actuations over the smart city elements.

**Figure 12:**
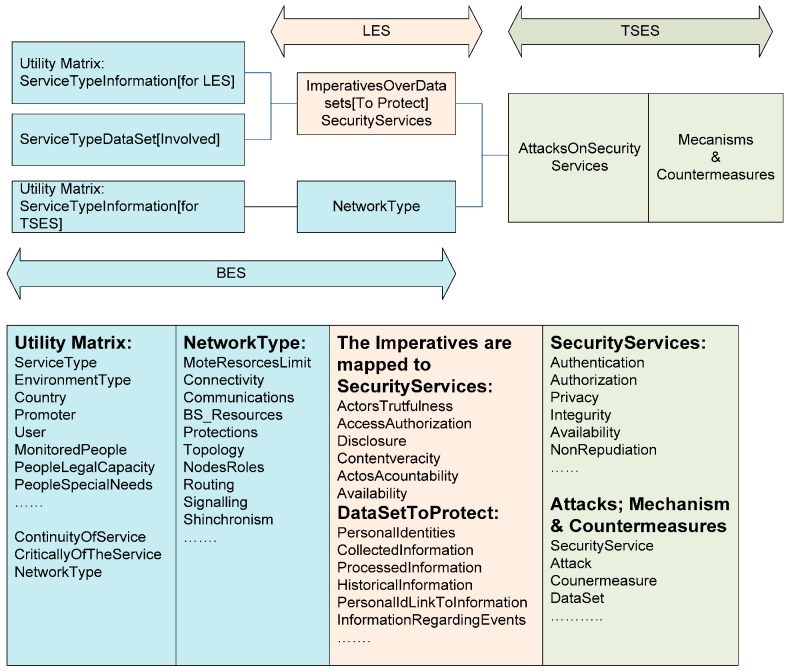
The most important information to select the adequate security and privacy mechanisms.

Respecting the knowledge network in the expert system, a general overview will be given in Figure 12 and is discussed in the next subsections, where the knowledge network in the system is described.

#### 5.3.2. Business Expert System (BES)

As shown in Figure 12, the BES knowledge is composed of the service type information, the data sets that could be sensitive (BES is not sure yet if those data sets must be protected or not), and finally by the characteristics of the type of used network for this service or IoT.

The service type information is stored in the “Utility Matrix” and is composed by two parts. The first one is composed of the information of interest for the processing of legislation in the LES, that is, the necessary data to select the legislative framework for the IoT service. It is composed by information such as the type of service, the type of operating environment, and the country where it is located. It also has the Information about the promoters, users and monitored entities (if they are people, their capacity and special needs are also known). The data that is considered as sensitive are also included.

The requirements of the service, as well as the necessity of its continuity, its own criticality, and the type of network that will provide it are also included. This is precisely the part that complements the utility matrix; it is the technical information about the type of network that is relevant when selecting among the security mechanisms in the TSES. The structure of the service, its own safeguards and bug handling, the possibility of operating the nodes as standalone, signaling and synchronization, monitoring interval, network segments, transmission sharing, information aggregation and routing are also stored.

#### 5.3.3. Legal Expert System (LES)

As shown in Figure 12, the LES knowledge is composed by the information about the legal imperatives to protect the data considered as sensitive by the legal framework. LES receives data from BES to compose the DPIA-T if does not exist yet.

As the LES processes information, data sets are put together, grouping personal and other sensitive data handled by the IoT service. A specific normative is applied to these data sets, assigning legal constraints or a necessary level of protection for a certain service type.

The service type is a function of the environment, country, promoters, users and monitored entities and their relevant legal characteristics. Actually, this information framing is coherent with the legal analysis that the NIST as well as the European Union carry out in their DPIA-T impact assessment templates.

If there is a set, {s_i_}, of services that possess infrastructures, and there is a set, {s_j_}, of joint services composed by subsets of {s_i_}, then there will be (i + j) impact assessment templates. It may also be the case that new services can be generated from elements of {s_j_}, by themselves or by combining with other elements of {s_i_}. In conclusion, the simplest approach is to have an impact assessment for each individual service, and one for each joint service, adapting the necessary mechanisms to each service. That way, services with an associated infrastructure are not hindered in regards to resources, achieving a tailored security. All data processing in LES is compatible with the “Data Protection Risk Assessment (DPIA)”.

#### 5.3.4. Technological Selection Expert System (TSES)

As shown in Figure 12, the TSES knowledge is composed by the security service information, and the information about the attacks, mechanism & countermeasures. The TSES processes information by mapping the imperatives in security services that need to be applied to data sets. These security services for a certain network type (resources, connectivity, communications, resources in the base station, topology, nodes, routing, signaling, and synchronism) are threatened by a list of attacks that affect the IoT service. These attacks have countermeasures (security service, network type, attacks, mechanism & countermeasure).

Luckily, nowadays there are already securities and privacy mechanisms that make it possible to use complex cryptographic mechanisms, supported by the rapidly advancing development of the hardware and operating systems of network elements. Currently, it is possible to cover the majority of cases routinely presented, and luckily there is a lot of activity focused in the creation of new solutions.

For the purpose of this paper, the security and privacy policy is considered as the set of mechanisms integrated within the services. The security policies and mechanisms’ suitability analysis is selected by the degree of coverage reached while having sufficient mechanisms, and by determining the best ones for the resources of the technology to which they are applied (delay, consumption, *etc.*). If not enough mechanisms are found, a coverage alarm would be set off in the corresponding knowledge base, urging the corresponding experts to solve the issue.

Attacks can be done by both outsider and insider attackers. Insiders, however, are able to perform worst damaging actions since they used to have a higher level of permissions and system knowledge.

If an overview of insider attacks is done, it can be noticed that, for instance, SCADA systems are used in many critical infrastructure applications that have important software components, such as Human Machine Interfaces, servers with historical data, Remote Terminal Units and the communication links between them. The latter include the units used to collect information and transfer it back to the central site carrying out any necessary analysis and control, and displaying that information on an operator screen afterwards. The operators use the Human Machine Interfaces data to make supervisory decisions. Therefore, the Human Machine Interfaces, data historians, communication links, sensors threshold values and actuator normal settings [36,37] can be attacked by an outsider (the attack can be initiated from outside, by unauthorized or illegitimate users; those usually are opportunistic, deliberate, and malicious) or by insider attackers (they happen when an authorized user misuses the permissions and damages the system by sending legitimate control commands with a great impact and higher success rate; these are difficult to predict and provide protection against them). With regards to users, not only engineers (responsible for managing object libraries and user interfaces, setting grid topology, normal work condition states, setting parameters of devices, defining process set points, writing automation scripts, *etc.*) have to be considered in a secure deployment, but also operators (expected to monitor the system status in Human Machine Interfaces servers, react to alarms and some events so that the process will run correctly, execute operator commands that often prevent triggering new alarms, resolve incoming alarms, make decisions about changing topology, *etc.*). An engineer is a more powerful system user than an operator, but the transmission system is controlled by operators. Insider attacks [36,37] to be considered are:

- Unresolved alarms attacks, when alarms are not perfectly resolved (delaying or making incorrect or incomplete actuations). These situations can provoke cascading failures of major consequences if a critical security error is unnoticed. Malfunctions sometimes are a consequence of wrong, high-level management decisions, such as budget cuts, transforming the activity of human operators from one of specialized nature to a multifunctional one without enough training, *etc.*
- Misconfiguration attacks of differing nature: Overload attacks (wrong changes of topology and load transfers, which can cause overload or a power failure in a large area), Outage attacks (opening the output feeders), incorrect setting attacks (improper equipment settings which could cause equipment incorrect operation).

The incident response can be a complicated matter because one minor mistake may result in the loss of the most critical pieces of evidence and make the whole case inadmissible for a trial or other court actions. Some mechanisms against insider attacks has been mentioned and referenced in [36,37,38,39,40,41], such as:

- Detect anomalous behavior in SCADA network traffic.
- Detect anomalies based on validating protocol specifications
- A real-time anomaly detection system for unknown attacks
- Anomaly detection for insider attacks based on system logs of the SCADA system to be periodically monitored to detect anomalous behavior (it is necessary to control time periods, parameter values, content of the command orders and many more variables).
- Detecting insider attacks in SCADA by data passing through the system and include a semantic module capable of understanding user actions.
- Statistical Anomaly Detection Method (SADM) is developed in some SCADA systems, by analyzing statistical properties of alarms that will determine the normal system behavior. SADM uses statistical properties to determine whether “current behavior” deviate significantly from the “normal behavior” by using the mean and standard deviation parameters in order to set thresholds, which can be learned from observations (operator behavior).
- Reference [42] talk about any malicious behavior changes statistical properties of alarms and is identified as an anomaly (experimental scenarios have been simulated by the proposed Colored Petri Nets (CPN)-model for insider attacks).

### 5.4. Processing Stages

Table 2 shows the basic process of selection and the knowledge bases involved. This operation mode enables the cooperation between experts from the areas involved through knowledge generated and formalized.

Simplicity was given a priority after numerous concept tests, so that the expert system is fast and user friendly. But this is not free, the coverage and reliability of policies and security mechanisms are transferred to the performance, coverage and reliability of the information in the knowledge bases of each of the three parts, BES, LES and TSES.

**Table 2:** Processing stages.

Information Involved		
Input	Knowledge Base	Output
**1**. Services requirements	Business Knowledge	**2**. Utility matrix & Personal data involved
**2**. Utility matrix & personal data involved	Laws and standards Knowledge	**3**. Legal Imperatives over sensible information
**3**. Legal Imperatives over sensible information	Attacks, security services, mechanisms Knowledge	**4**. Security services & mechanisms over information pieces
**4**. Security services & mechanisms over information pieces.	Business Knowledge	**5**. Final decision
**5**. Final decision	Validity check	**6**. Legal certification

*Utility matrix* & *personal data involved* are made in a Business Expert System (BES) based on the final service requirements and the information managed. The user provides all this information through forms, in a guided way. The utility matrix is composed of two parts. The first part is comprised of the information about the type of the final service, about the country, the developers and users, the entity being monitored (persons, animals or things), the characteristics of the persons subject to monitoring (children, adults, seniors, their legal capacity, special needs, *etc.*). With this information and the data involved in the service (some of them may be personal data) the Legal Expert System (LES) is able to perform the necessary processing to obtain the legal requirements that must be implemented in order to protect the information which must be protected. The second part contains more technical information about the network type, the sensor nodes resources, the base station and the connection types, the communication used, network topology, type of routing, signaling, synchronism, if continuity of service is required or not, and the level of service criticality (critical for people, for infrastructures, *etc.*). These data are needed by the Technological Selection of security solutions Expert System (TSES) to determine the possible service vulnerabilities.

The correct selection of security services and mechanisms strongly correlates with the amount of information available about technology, topology and information extracted from the Utility Matrix [5,6]. It is clear that not all technologies are able to support all the existing mechanisms without affecting quality of service. With the current knowledge available about the technological possibilities, it may be possible to form a synergy between the security services and mechanisms in order to obtain the minimum processing for the security and privacy required.

Laws and standards Knowledge: The LES knowledge base does not store laws; it stores the knowledge of experts in their area about the legal requirements to apply to personal data on the final service. The legal framework is obtained from the extracted information from the Utility Matrix (service type, country, environment type, if continuous monitoring is needed, or if this is a critical service or not). From this legal framework legal imperatives are extracted. Legal requirements are identified by the main concept represented:

- Actors’ truthfulness is transformed into “Authenticity”;
- Access authorization is transformed into “Access control”;
- Disclosure or dissemination of information is transformed into “Privacy”;
- Content’s truthfulness is transformed into “Integrity”;
- Actors’ responsibility is transformed into “Non-repudiation”;
- Availability and continuity of service is transformed into “Availability”.

The data that may need to be protected are those that identify to the people individually and are related to their gathered data, their processed about historical data, or the complete events that identify the state or situation of the person, *etc.*

The conceptual direct relationship is established between *the LES* legal imperatives obtained and security services by Recommendation X.800 [43] in TSES. The attacks on the final service are countered by the security services which comprise countermeasures and security mechanisms. In the TSES knowledge base the security services, and the countermeasures and the security mechanisms are associated with the attacks. The obtained result is a set of mechanisms and countermeasures to achieve the security level needed. When the system is unable to find a solution for all requirements of security and privacy, a warning of insufficiency of knowledge is thrown indicating the problem encountered.

One part of the assigned work to the technological human experts of TSES is to feed the knowledge database with useful mechanisms that may be utilized. These mechanisms will be classified according to several features concerning the casuistry where they are successful when applied, their effectiveness against attacks (based both on a study made for this solution and past experience), an assessment of easiness of change and adjustment and the capacity of the each mechanism to be monitored.

The ACCUS platform directly supervises the involved security mechanisms, attack attempts and the successful attacks, and once the attack has been mitigated, the involved mechanisms will be revised, and corresponding changes are made in the platform, in the service and in the expert system.

### 5.5. Performance Evaluation

The expert system is responsible for controlling the level of current legislation fulfilment regarding asset protection (that is, the level of fulfilment of legal obligations). The smart city offers a collection of individual services prone to be attacked. Each of the services is subdued to some specific “legal obligations” that are implemented by means of “countermeasures and security mechanisms”. “Attacks” may cause an “impact” on the assets that must be protected, so the level of fulfilment regarding current legislation is assessed on the basis of the impact that has taken place (“0” = no impact registered, “1” = impact on non-legally protected assets, “2” = impact on legally protected assets). There are three indicators:

- No legislation-based impact attacks: (Service; Attack; Impact (0 U 1)).
- Seriousness of the impacts: (Service; Attack; Impact (0, 1, 2)).
- Legislation-based impact: (Service; Attack; Impact (2)).

These indicators can be aggregated to the overall service or disaggregated in individual services from the values: V_n’, k’_ (Indicator value for indicator k’, for the service n’). When impact is mentioned, it is referred to one value k’ ∈ [1, k] depending on the specific service n’ ∈ [1, n] it has been aggregated to. Value V is the one corresponding to the impact.

Thus, the number of attacks without legal impact, the number of attacks with legal impact, and a quantitative measure of the seriousness of the occurring impacts can be known. It is also possible to know which the most attacked services and the attacks that cause greater impact are, and therefore assess the mechanisms against the attacks.

To find the main causes that lead to unwanted impacts, auxiliary measures must be obtained to establish an improvement plan. These measures are obtained from the service platforms that can send them, or logging in periodically and processing this information from their event logs. These indicators are basically two: (1) Effectiveness of the mechanisms and countermeasures against security attacks and (2) Overhead introduced by those mechanisms in the system when these mechanisms are operating and may affect the quality of the service (traffic, delays and excessive resource consumption), *i.e.*, Overhead caused by defensive actions.

This performance control is executed by the expert system, based on an alarms system which is described below:

- Security system states:
  ○ All Seem Well (ASW), no alarm condition.
  ○ Alarm categorized as “Minor alarm”, “Major alarm” or “Critical alarm”.
  ○ An “Alarm Ceasing” condition appears when the alarm condition disappears.
- There is an alarm increasement-related category policy because of alarm accumulation and the usage of an alarm decreasement category policy if there is no alarm repetition in a certain time interval.
- When there is not any alarm the text ASW appears, and the Minor, Major and Critical alarms text with green background. When alarms appear the Minor background is blue, the Major background is yellow, and the Critical background is red. If we look at the panel and on the alarm background the number of detected alarms in each category appear highlighted. For example, supposing that in one moment there are 3 “Minor” alarms and 1 “Major” alarm, number “3” is highlighted on the Minor alarm background and number “1” on the Major alarm background. All the data of the alarms, their activity, their start and the alarm ceasing, are stored in the alarmlog. Based on the alarmlog information the statistics about the alarms are established.

This concentration of alarm information gives the possibility of generating higher-level alarms and proactive alarms. When an alarm is received, is categorized with an alarm level based on the knowledge base (V_n_,_k_; L_Alm_). After that, its alarm level (A_n_,_k_) may change according to the number of repeats and time interval. Each one of the attacks on a service is associated with a mechanism, along with the impact. Noting "the time until the alarm ceases", "the associated mechanism" and the "code of completion of the action" for a mechanism, a general vision is obtained. The expert system also evaluates the features regarding the properties of the mechanisms which are:

- Flexibility: the simplicity (or complexity) degree of change and readjustment regarding security mechanisms, either due to a change in legislation or an update in the smart city alert level (pre-emptive actions, natural disasters, *etc.*) is considered too. if because of one of these reasons security levels in a smart city have to be modified, the ability of modifying them with ease (even remotely if possible) will improve the efficiency of the smart city.
- Capability of being monitored: this parameter measures the capacity of one security mechanism to be monitored. Manufacturers may provide tools to supervise the procedures that they have enabled for their equipment. If this is not the case, they must be developed by human experts and described in TSES.
- There is also knowledge coverage control over the knowledge in the BES, LES and TSES, which usually gives way to revisions and adjustments of the mechanisms.

All this information is analyzed and then enters the system improvement plan.

As a conclusion of the performance evaluation, the main intangible benefit is to provide fast answers to new legal risks about personal data protection in the IoT environment, and these answers are provided by the entity capable of doing so (juridical area) and control the results. Another benefit is to allow companies to conduct cost studies and test ideas for new products and services before beginning the development process.

The main tangible benefit is to provide a tailored security and privacy implies that use the necessaries mechanisms only. It represents as results to obtain savings in resources for sensor nodes. In some cases these savings avoid to use heterogeneous sensor network, for example. If a promoter want to provide the same service such as “health monitoring” for different user types (people, animals, or plants), each one has a different requirements on privacy. These savings can be calculated by the following expression (Equation (1)):

(1) $$\text{X}\cdot \text{Y}=\text{Z};\;\left(\begin{array}{ccc} b11 & . & b1k\\ . & . & .\\ . & . & .\\ bn1 & . & bnk \end{array}\right)\cdot \left(\begin{array}{c} SecService1\\ .\\ SecServicek \end{array}\right)=\left(\begin{array}{c} SecServices\;UserType\left(1\right)\\ .\\ .\\ SecServices\;UserType\left(n\right) \end{array}\right)$$

X represents the assign matrix; Y is the matrix that represents the possibilities to provide security services for one specific technology in the final service, finally Z is the set of services assigned to one user type.

Matrix X assign the security services to user type. Each row represents the services for one user type and the each column enable “1” or disable “0”, the specific security services, so b[nk] is the enable o disable value for the security service “k” for the user type “n”, SecServices UserType(k) is the set of security services assigned to the user type(K).

All users have to belong to the defined user types, and each user type has a certain percentage of users inside. For example, suppose that 25% of users belong to each of the four groups; SecServices UserType(1): cows; SecServices UserType(2): horses; SecServices UserType(3): footballers; SecServices UserType(4): firefighters, according with [6], the expression is Equation (2):

(2) $$\left(\begin{array}{cccc} 0 & 0 & 0 & 0\\ 1 & 1 & 0 & 0\\ 1 & 1 & 1 & 0\\ 1 & 1 & 1 & 1 \end{array}\right)\cdot \left(\begin{array}{c} Authenticity\\ Privacy\\ Integrity\\ \text{Availability} \end{array}\right)=\left(\begin{array}{c} No\;Sec\\ Aut+Priv\\ Aut+Priv+Int\\ Aut+Priv+Int+Avail \end{array}\right)$$

25% of users do not have any security services, and the others save some services. Each service can be quantified in spent of resources in terms of energy, delay, dollars, *etc*. Another tangible benefit is that by concentrating the intelligence in a NOC, not only leads to greater specialization, the manpower costs are rationalized in a coherent dimension.

## 6. Application Scenario for a Smart Service Providing Security, Privacy and Trust: “Living at Home Longer, Autonomously and Safely”

The goal was to develop a service for elderly people named “Living at home longer, autonomously and safely“. This service is composed of one service called “Enhanced tele-assistance at home” and another one called “Safe home”. The next paragraphs describe the security and privacy requirements, as well as the solutions proposed and the performance evaluation.

The first service, named “Enhanced tele-assistance at home” has been designed to specifically address the requirements of the elderly. It is composed by a gateway at home connected using land and mobile lines. It supports several protocols, such as TT21 (dual tone multi-frequency signaling or DTMF, and the sequential/single tone multi-frequency (STMF) protocol for mobile GSM/Next Generation Networks (NGN) and Telecare Home Units), TT92 (DTMF and STMF), BS8521 (DTMF), TTNEW (DTMF) so as to send/receive calls to/from the assistance center. In the home subsystem, the gateway is connected through the sink to a wearable body sensor network (ZigBee) in order to retrieve the body temperature, heart rate, and fall detector events. The system has been designed to monitor several concurrent users in the same home; all these data are sent to the assistance center through the Internet.

The second service is named “safe home” and it is addressed to a wide range of users. This service is composed by a gateway at home connected to the assistance service through the Internet. In the home subsystem, the gateway is connected through the sink to several sensors deployed throughout the house (ZigBee) providing security alarms related to the indoor temperature, CO, smoke, gas, water flood, as well as events related with open doors or windows. Those alarms can be audible and addressed to an attention center to receive assistance. When a CO, smoke or gas alarm is triggered, the actuators close the corresponding latch and open the windows.

The composite service: “Living at home longer, autonomously and safely” is basically composed of both mentioned services, with some changes in several sensors, bearing in mind that this composite service is designed for elderly people. The person’s health and the living environment must be monitored. It is composed by the same gateway mentioned in the first service, with the capacity to also manage the second service using the same gateway and sink.

### 6.1. DPIA-T for This Service

Since there is no Data Protection Impact Assessment-Template (DPIA-T) related with this service, the expert system has to do the processing stages mentioned in Table 2. As said before, a composite service has its own security and privacy requirements, and can have substantial differences regarding the individual services. Let us see the utility matrix in order to obtain the security and privacy requirements (Table 3 and Table 4).

**Table 3:** Utility Matrix: Service.

Utility Matrix:	Description
Service name	Living at home longer autonomously and safely.
Service Type	Health-care; Safety
Environment Type	Home
Country	Spain
Promoter	Joint venture: Health care and Home insurance companies
User	Elder people
Monitored person	People and rooms at home.
Legal capacity of person	Full legal capacity
Special needs person	Elder people with logical limitations, without special needs
Continuity of service	Push button, critical sensors for life: CO, smoke, gas, presence sensor and outside door and windows open and critical sensor for service.
Critically of the service	high
Network type	NW_Type1

The network type must be defined also.

**Table 4:** Utility Matrix: Network type for the service.

Network Type:	Living at Home Longer Autonomously and Safely
Network Type Name	NW_Type1
Mote resources limit	Wearable mote: Memory to store data on standalone operation
Connectivity	Radio
Communications	Wearable mote—Gateway, via radio when push button is pressed to call with assistant center. ZigBee connection between wearable node and sink for send data via internet to the assistant center.
	The home sensors—sync via ZigBee and connection via internet from gateway to service provider. In case of CO, gas or smoke alarm, is communicated to actuators to shut down the problem and open outside window and send alarm to the person.
BS Resources Limit	None, when power is down, it has batteries and connections via GSM, 3G. In home there is an emergency battery for four hours (emergency light and sensors power).
Topology	Star
Nodes Roles	The wearable node has collected basically function
	All nodes has collected basically function except window sensor node; it has an actuator function to open outside window directly when CO, gas or smoke are detected.
Routing	Routing is unicast for all sensors to Gateway.

Security imperatives in DPIA-T Format, according to the Spanish (and European) legislation on personal data protections are as follows [6]:

- Data related health must be protected or at least unlinked from the personal identity.
- Data must be fresh and true.
- The data related to the intimacy at home must be protected.
- Critical data for life safety are a priority.

The legal imperatives over sensitive information are structured in a DPIA-Template Security service-Attacks-Defences type format (Table 5) in the case of our current service. Now is the time to assign the specific protections to the data set of the composite service (Table 6). There are several changes compared with the individual services: a water flood can cause a fall, so it is considered as a critical sensor in this composite service, as well as the presence in home and presence in bed. These are important pieces of information so as to provide a good service for elderly people.

**Table 5:** Legal Imperatives in DPIA-T format.

DPIA-T: Living at Home Longer, Autonomously and Safely			
Security Service	Attack	Target	Defence
Availability	DoS	1) The physical layer is degraded and the communication among nodes is impossible (jamming).	The situation must be known to face it.
		2) A spurious node starts sending malicious data packets to the network.	
Authentication	Sybil	A node is asking for multiple IDs, and if the attack succeeds, the node is able to subvert the trust mechanism.	Restore trust mechanism by rejecting the malicious node.
	Node replication	When a node ID is copied, replicated in a new node, and then introduced in the network. From that moment on, the network accepts the node with the cloned ID as an authorized node.	Realize and revoke the malicious node.
	False node	It introduces data traffic in the network to stop legitimate nodes from communicating (injecting false data messages, requesting authorization continuously, *etc.*).	Identify the false node and discard all messages.
Integrity	Message corruption	When a message reaches the recipient with a different content than the one sent by the source. This situation is either because the message has been degraded in the transmission, or because the message has been intercepted and intentionally changed.	Ensure that messages have not been altered.
Privacy	Eavesdropping	Other devices listening in the same frequency may intercept all communications between two nodes.	Provide authentication and ciphering capabilities.
			Use data anonymization.
	Node subversion	When a node is captured and cryptoanalyzed the secret keys, node ID, security policies, and so forth are disclosed.	Use few data stored in each node and renew the keys.

**Table 6:** Data protection over data sets.

Sensor		Reason	Tipo	Auth	Integr	Privacy	Avail	Intruders Insiders
1	Push button	Emergency	Body	-	-	-	Y	-
2	Temperature	Private information		Y	Y	Y	Y	Y
3	heart rate							
4	Fall detector							
5	Temperature	Auxiliary Information	Home	Y	Y	-	Y	
6	CO	Vital for life				-		
7	Smoke					-		
8	Gas					-		
9	Water flood					-		
10	Door	Vital for security				Y		
11	Window							
12	Presence	Vital for Service						
13	Pres in bed							

### 6.2. Security Services and Mechanisms

Once the data set protections of the composite service are assigned, the system must look for the appropriate mechanisms in the knowledge base to face the attacks mentioned in the DPIA-T. For these types of service and network, as shown in Table 4, the network type is a condition required to choose the mechanism to be used. In this case, the parameter “mote resources limit”, has the value “wearable mote”, and for these network types (in this case the mechanism must be lightweight) TSES selectw the SensoTrust proposal [44] mechanisms. All used mechanismw in this example are able to notify when an incident occurs. Each mechanism is evaluated, and assigned a value in the “Past experiences” parameter “0” non effective mechanisms; “1” effective mechanism, it can be monitored, manual reactions; “2” effective mechanism, it can be monitored, automatic reactions. This is based on the trust domains definition where each of them has a common security policy. In this case the domains are defined as follows: as we can see in the previous table, there are two major types of security and privacy required, for each one a domain is defined as shown in Table 7. “Push button” is out of domain because when the button is used the station makes a call outside the WSN.

**Table 7:** Trust domains defined.

Sensor		Trust Domain	Sensor Type
1	Push button	Out of domains	Wearable
2	Temperature	Domain 1 Policy	
3	heart rate		
4	Fall detector		
5	Temperature	Domain 2 Policy	Home
6	CO		
7	Smoke		
8	Gas		
9	Water flood		
10	Door	Domain 1 Policy	
11	window		
12	Presence		
13	Presence in bed		

After applying the legal imperatives (DPIA-T) to the current case, the following list of security and privacy mechanisms arises, taking into account that it can be applied as the common scheme indicated (key distributed, roles and trust policies) in SensoTrust [44,45]. Each domain has its own security and privacy policy as is shown in Table 8 and Table 9.

**Table 8:** Trust domain 1 policies.

Domain 1 Policy		
Security Service	Attack	Countermeasure
Availability	DoS	**Mech_DoS_1**: One alarm is triggered in the Security Manager informing about the situation
Authentication	Sybil	**Mech_Sybil_1**: In the security scheme, every node ID is preconfigured for each node and only the Security Manager (out of the WSN) has the complete list of the IDs. *In extremis*, it is possible to perform a node revocation.
	Node replication	It provides two mechanisms to avoid this attack.
		**Mech_N_Repl_1**: The Node ID is stored in an external entity (SM) that controls all the IDs working in the network.
		**Mech_N_Repl_2**: Security policy, if the SM detects that two nodes are operating with the same ID, a node revocation protocol is issued, and the node is dropped from the network.
	False node	**Mech_N_False_1**: Using the node ID, the schema is able to identify the false node and, using the domain key renewal functionality, all the messages sent by this node will be discarded.
Integrity	Message corruption	**Mech_Msg_Corrupt_1**: To avoid both issues, security schema includes the ciphering suite functionality, which allows performing a message hash (using MD5, SHA1, *etc.*).
Privacy	Eavesdropping	**Mech_Eavers_1**: To avoid data disclosure, it provides both symmetric and PKI ciphering capabilities.
		**Mech_Eavers_2**: Anonymization, unlinking the personal identification and his/her measure data
	Node subversion	**Mech_N_Subv_1**: To avoid it is to minimize the cryptographic and security information stored in each node. Nevertheless, all the keys in the network can be renewed.

**Table 9:** Trust domain 2 policies.

Domain 2 Policy		
Availability	DoS	**Mech_DoS_1**
Authentication	Sybil	**Mech_Sybil_1**
	Node replication	**Mech_N_Repl_1**
		**Mech_N_Repl_2**
	False node	**Mech_N_False_1**
Integrity	Message corruption	**Mech_Msg_Corrupt_1**

Countermeasures against outsider attacks are based on authentication, and the countermeasures against insider attacks are based on the security policies and the trust domains.

### 6.3. Performance Evaluation

To preserve the Quality of Service (QoS) it is necessary to know the limitations (Table 10). In the sensor nodes used for the testing purposes, the maximum power computation was limited below 20%, since it was considered that 20% of this maximum value is able to ensure proper operation. With Sybil and False node, the node load is keeping below the maximum limit defined.

**Table 10:** Restrictions and limitations.

Sensor		Critical Requirement	
		Battery	Delay
1	Push button	Y	N
2	Body Temperature		Y
3	Heart rate		
4	Fall detector		
5	Home Temperature	N	N
6	CO		Y
7	Gas		
8	Smoke		
9	Water flood		
10	Outside door		N
11	window		
12	Presence at home		
13	Presence in bed		

**Figure 13:**
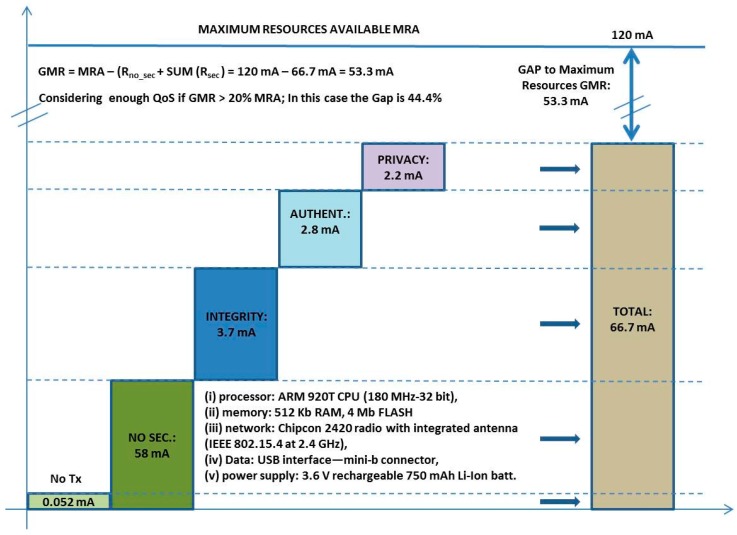
Energy spent *vs.* security services.

**Figure 14:**
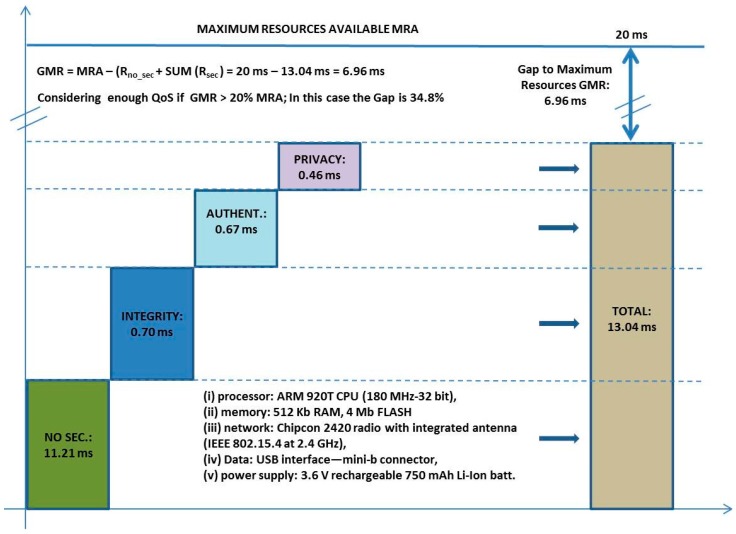
Delay *vs.* security services.

The battery life is only important in case of Wearable devices, because gateway, sink and devices in home have battery for emergency light and sensors with enough autonomy when the electric power is fell down. The results have been obtained in laboratory for the policy more restrictive (Figure 13 and Figure 14). Finally, it is necessary that the system is designed to provide reports about both the anomalies found (true + and −, false + and −) as their reactions.

Reporting to BES and TSES:

- No legislation-based impact attacks.
- Seriousness of the impacts.
- Legislation-based impact.
- Alarm report

Reporting to LES:

- Legislation-based impact.
- Improvement plan.

Respecting delays only wearable sensors, CO, Gas and smoke sensors are important while the communication with the windows actuator is connected by wire.

Some indicators can be [46]:

Percentile 90: The time until to solve the faults.
Considering MTBF: Mean Time Between Failures (true positives + false negatives) and MTTR: Mean Time To Repair (true positives + false negatives + false positives).
MTTR over false positives represents the resources spent on inefficient results.
The actuations on false negatives represent the impact when a problem is not detected on time.
Coef (no attacks) ≥ MTBF/(MTBF + MTTR).
Regarding the system behavior, it has obtained the following results (Table 11 and Table 12).

**Table 11:** Citizen protection impact and reaction.

Impact and Reaction:				
Security Service	Incidences	Impact	Resolution Time	Pending
Authentication	7	1	Manually	0
Integrity	7	1	Manually	0
Privacy	7	2	Manually	0
Other incidences	-			

These used mechanisms in the application scenario only notify about the problem, and the corrective actions are manual. Resolution time only appear for automatic actions. Respect to the operation of the entire system, the results were as follows.

**Table 12:** System parts behavior.

**BES: Users Perspective. Forms Validation**			**BES-USER interaction**
*Validated*	*Rejected*	*Validation time*	
10	4	70 ms	
**BES: Making Utility Matrix 1st Part (Service)**			**Process and BES-LES interaction**
*LES validations*	*LES rejections*	*Process time*	
6	0	4.2 s	
**BES: Making Utility Matrix 2nd Part (Network Type for the Service)**			**Process and BES-TSES interaction**
*TSES validation*	*TSES rejections*	*Process time*	
6	0	3.2 s	
**LES: Validation Utility Matrix 1st Part**			**Internal Process only**
*Validated*	*Rejected*	*Validation time*	
6	0	120 ms	
**LES: Making DPIA-T**			**Process and LES-TSES interaction**
*TSES validations*	*TSES rejections*	*Process time*	
6	0	3.5 s	
**TSES: Validation DPIA-T**			**Internal Process only**
*Validated*	*Rejected*	*Validation time*	
6	0	30 ms	
**TSES: Validation Utility Matrix 2nd Part**			**Internal Process only**
*Validated*	*Rejected*	*Validation time*	
6	0	50 ms	
**TSES: Making Policies**			**Process and TSES-BES interaction**
*BES validation*	*Rejected*	*Process time*	
6	0	7.8 s	
**TSES: Making Policies**			**Process and TSES-ACCUS interaction**
*ACCUS validation*	*Rejected*	*Process time*	
6	0	12.1 s	
**Service Platform: Policy Validation**			
*Accepted*	*Rejected*	*Validation time*	Internal process. is possible to do it with the information received?
6	0	340 ms	
**Service Platform: Actions Generated**			
*Actuations completed*	*Time to complete*	Generate the actuations and configure the testing nodes.	
6	18.21 min		

Ten services have been perfomed for this test, four of them had bad data, and the other six were well done. Times are measured on viable services, because rejections are much faster. Interactions include processing and the information transfer between them.

### 6.4. Providing the Obtained Results to the ACCUS Platform

Once the mechanisms have been selected, they must be provided to ACCUS platform in order to continue the process and configure the service. The following lines present the process to communicate the policy corresponding to trusted domain number 2.

The selection criteria of security mechanisms are as follow for trust domain 2. The input and output xml can be:

/*Input Model*/
<?xml version=*"1.0"* encoding=*"UTF-8"*?>
<in:input xmlns:in=*"http://www.grys.org/securityServicesIN"*>
  <in:contextOfProtection>Smart Home  Trust Domain 2</in:contextOfProtection>
  <in:facestToProtect>
    <in:facet>  Temperature  </in:facet>
    <in:facet>  CO  </in:facet>
    <in:facet>  Smoke  </in:facet>
    <in:facet>  Gas  </in:facet>
    <in:facet>  Water Flood  </in:facet>
  </in:facestToProtect>
</in:input>

/*Output Model*/
<?xml version=*"1.0"* encoding=*"UTF-8"*?>
<out:recommendations xmlns:out=*"http://www.grys.org/securityServicesOUT"*>
  <out:services>
    <out:service>
      <out:name id=”av”>Availabilitu</out:name>
             <out:mechanism> 
                   Mech_DoS_1
             </out:mechanism>
    </out:service>
    <out:service>
      <out:name id=”auth”>Authentication</out:name>
             <out:mechanism> 
                   Mech_Sybil_1
             </out:mechanism>
            <out:mechanism>
                   Mech_N_Repl_1 
                   Mech_N_Repl_2
             </out:mechanism>
             <out:mechanism> 
                   Mech_N_False_1
             </out:mechanism>
    </out:service>
    <out:service>
      <out:name id=”int”>Integrity</out:name>
             <out:mechanism>
                   Mech_Msg_Corrupt_1 
             </out:mechanism>
    </out:service>
  </out:services>
  
  <out:attacks>
    <out:context> Smart Home</out:context>
    <out:attack refTo=”av”>
      <out:description> DoS </out:description>
    </out:attack>
	
    <out:attack refTo=”auth”>
      <out:description> Sybil </out:description>
      <out:description> Node replication </out:description>
      <out:description> False Node </out:description>
    </out:attack>
	
    <out:attack refTo=”int”>
      <out:description> Message corruption </out:description>
    </out:attack>
    
      <out:secSerToImplement> 
      		Availability Authentication Integrity 
      </out:secSerToImplement>
  </out:attacks>
</out:recommendations>


All of this effort has secured only one smart service in the city. However, this process should be iterated for all possible service combinations.

## 7. Conclusions and Future Work

Collaboration between multiple areas (business, legal and technological) is critical in order to provide users with the necessary trust in the security of the service and protection of their personal data. The limitation of resources of wireless sensor networks for products and services makes it necessary to implement a tailored security schema to avoid the risk of poor quality of service when resources are exhausted. In this way, the intelligent combination of security mechanisms available could increase the service’s efficiency.

In an environment where several services and technologies coexist, the intelligence of the decision-making on security policies could be integrated into the officially recognized and certified network operations centre which would provide a large capacity of management, updating the deployed security measures. Since there is no legislative uniformity, it is necessary to develop tools and methods that permit a transition period with minimal risk to people and their rights.

When citizens’ quality of life greatly depends on the correct performance of smart cities, which, in turn, depend on the correct performance of systems and services (and the networks in which they are set up), it might be required to configure and reconfigure privacy and security policies. It may be due to changes in states of emergency, alarm, *etc.* Or, they may be necessary because of dynamic changes within the city. Therefore, it is important to know timely and in due form the security policies that must be implemented in each case, in a reliable way, by the expert system described. It would also be convenient to deploy and perform the actions needed on the elements of the city, fast and efficiently (decreasing human intervention to a minimum), using the mediation chain described. The key lies in the cooperative environment between the three main knowledge areas described, and in a common management, even if the systems are technically duplicated or diversified to augment security and availability.

It must be considered that, in an uncertain future, a failure in the performance of a smart city may be disastrous and could have serious consequences; far from just a mild annoyance, it could result in the endangerment of people and infrastructure. A service under attack or functioning incorrectly may activate an alarm, with no emergency triggering it. Therefore, investing in security, privacy and reliability in the performance of systems in a smart city may be worthwhile.

In the future, we will point in different directions according to the road map. On the expert system side knowledge bases should be enhanced to make possible interactions between IoT platforms and several technologies. Also is will be necessary to make life easier for the human experts providing them nice tools to manage the information. ACCUS must go on with its road map, to improve the functionalities to create new services in the smart city environment. Those new services represent good opportunities for both systems to work together to face new issues.
